# Editorial: Advances in Nucleic Acid-Based Biosensors and Imaging

**DOI:** 10.3389/fchem.2022.925082

**Published:** 2022-05-24

**Authors:** Jane Ru Choi, Mithran Somasundrum, Muhammad J.A. Shiddiky, Werasak Surareungchai, Yufang Hu, Zhihe Qing

**Affiliations:** ^1^ Life Sciences Centre, University of British Columbia, Vancouver, BC, Canada; ^2^ Biosciences and System Biology Team, Biochemical Engineering and System Biology Research Group, National Center for Genetic Engineering and Biotechnology, National Science and Technology Development Agency at KMUTT, Bangkok, Thailand; ^3^ Queensland Micro and Nanotechnology Centre (QMNC), Griffith University, Brisbane, QLD, Australia; ^4^ School of Environment and Science (ESC), Griffith University, Nathan, QLD, Australia; ^5^ School of Bioresources and Technology, King Mongkut’s University of Technology Thonburi, Bangkok, Thailand; ^6^ Nanoscience and Nanotechnology Graduated Research Program, Faculty of Science, King Mongkut’s University of Technology Thonburi, Bangkok, Thailand; ^7^ State Key Laboratory for Managing Biotic and Chemical Threats to the Quality and Safety of Agro-products, State Key Laboratory Base of Novel Functional Materials and Preparation Science, School of Materials Science and Chemical Engineering, Ningbo University, Ningbo, China; ^8^ Hunan Provincial Key Laboratory of Cytochemistry, School of Chemistry and Chemical Engineering, Changsha University of Science and Technology, Changsha, China

**Keywords:** nucleic acid, DNA/RNA, sensing, diagnostics, imaging, detection

Nucleic acids are key biomolecules that regulate the expression of hereditary information within living organisms (Jani et al., 2019). The predictable and specific Watson–Crick hybridization of complementary bases of nucleic acids renders them extremely useful for biomedical applications including biosensing and bioimaging (Jiang et al., 2020; Ma and Liu, 2020). In recent years, functional nucleic acids, including molecular beacons, aptamers and DNAzymes have been synthesized in such a way that they can specifically bind to various analytes including metal ions, organic dyes, amino acids, oligosaccharides, toxins, enzymes, and cells (Choi, 2020). This key property has prompted the use of functional nucleic acid for the detection of targets based upon numerous detection approaches, including fluorescent, colorimetric and electrochemical detection (Hwang et al., 2020).

This Research Topic highlights the use of functional nucleic acids for fundamental research and applications. A number of comprehensive review articles have highlighted the recent advances of functional nucleic acids and their biomedical applications. For instance, Yang et al. reviewed the advances and biological applications of DNA-templated silver nanoclusters (DNA-AgNCs). DNA-AgNCs, an emerging fluorophore, possesses unique features including high fluorescence quantum yields and stability, good biocompatibility, and low toxicity, making them highly suitable to be used as fluorescent probes. Their synthesis methods and biomedical applications, such as fluorescent sensing and imaging, were comprehensively reviewed. Dyussembayev et al. reviewed the advances in biosensors for detection and quantification of plant pathogens. The conventional methods used in plant disease diagnostics were compared with new nucleic acid-based biosensing technologies, especially electrochemical and optical biosensors for pathogen detection. The remaining challenges and future perspectives were briefly discussed. In addition, Huang et al. reviewed the applications of nucleic acid probe-based fluorescent sensing and imaging for cancer diagnosis and therapy. The characteristics of nucleic acid probes and their latest advances in fluorescent sensing and imaging were summarized, particularly in cancer diagnosis and therapy. Some challenges and perspectives in the field were also elaborated.

A number of Research Articles in this topic have reported the use of nucleic acid-based biosensors for fundamental and application research works. These studies have employed nucleic acids as recognition elements in biosensors. For fundamental studies, Wang et al. has reported the use of NiCo riboswitch-based whole cell biosensor to detect Co^2+^/Ni^2+^ transport in *E. coli* to study their cellular processes. In general, the NiCo riboswitch prevents formation of an overlapping intrinsic terminator and promotes production of full-length transcription product of mCherry when Ni^2+^/Co^2+^ is bound. This enables transcription of the reporter gene which produces fluorescent signal. This technology will be useful in monitoring the changes of intracellular concentration of Ni^2+^/Co^2+^ when investigating the transport mechanism using genetic deletions. Since the Co^2+^/Ni^2+^uptake of pathogenic bacteria shows a relationship to pathogenicity, the biosensor can be further applied to study bacterial infections.

Apart from that, several studies have reported the applications of functional nucleic acids, including the detection of single nucleotide polymorphisms (SNP) and mycotoxins. For instance, Xia et al. introduced a novel biosensor to detect SNP based on the quenching effect of fluorescence-embedded SYBR Green I dye (SG) and graphene oxide (GO). This biosensor is composed of GO and SG. SG is well embedded in fully complementary dsDNAs and produces high fluorescent signal. However, a single matching sequence (SNP) usually results in unstable DNA double helix, which exhibits poor SG embedding and low fluorescent signal. GO enhances the unwinding of unstable SNP through strong GO/ssDNA interactions, thereby quenching the fluorescence from free SG and SG/SNP. In another study, Qiao et al. reported the use of an aptamer-based fluorescence quenching approach for the detection of aflatoxin M1 in milk. In this biosensor, the specific aptamer was labeled with FAM (carboxyfluorescein), and their cDNA were labeled with a carboxytetramethylrhodamine (TAMRA) quenching group. In the presence of aflatoxin, a structural switch in the aptamer was induced by forming an aflatoxin/aptamer complex. This structural change led to the release of the cDNA, producing a fluorescent signal. The biosensor could be useful for high-throughput quantification of mycotoxin levels in dairy products. While the above-mentioned fluorescent detection methods require external fluorescence detectors, they are more sensitive than colorimetric detection methods such as colorimetric paper-based biosensors and lateral flow assays. The GO-based biosensor, in particular, requires no fluorescent labeling, which is much simpler and less expensive compared to the other methods.

In conclusion, this Research Topic provides an in-depth overview on functional nucleic acid-based biosensing and imaging. The functional nucleic acids offer enormous potential for guiding appropriate health care, playing a significant role in medical diagnosis and therapy, food safety and environmental monitoring.
